# Anomalous Perceptions and Beliefs Are Associated With Shifts Toward Different Types of Prior Knowledge in Perceptual Inference

**DOI:** 10.1093/schbul/sbx177

**Published:** 2017-12-27

**Authors:** Daniel J Davies, Christoph Teufel, Paul C Fletcher

**Affiliations:** 1 Department of Psychiatry, University of Cambridge, Cambridge, UK; 2 Cardiff University Brain Research Imaging Centre (CUBRIC), School of Psychology, Cardiff University, Cardiff, Wales, UK; 3 Cambridgeshire and Peterborough NHS Foundation Trust, Fulbourn Hospital, Cambridge, UK

**Keywords:** psychosis, schizotypy, predictive processing, perception, prior knowledge, computational modeling

## Abstract

Psychotic phenomena manifest in healthy and clinical populations as complex patterns of aberrant perceptions (hallucinations) and tenacious, irrational beliefs ( delusions). According to predictive processing accounts, hallucinations and delusions arise from atypicalities in the integration of prior knowledge with incoming sensory information. However, the computational details of these atypicalities and their specific phenomenological manifestations are not well characterized. We tested the hypothesis that hallucination-proneness arises from increased reliance on overly general application of prior knowledge in perceptual inference, generating percepts that readily capture the gist of the environment but inaccurately render its details. We separately probed the use of prior knowledge to perceive the gist vs the details of ambiguous images in a healthy population with varying degrees of hallucination- and delusion-proneness. We found that the use of prior knowledge varied with psychotic phenomena and their composition in terms of aberrant percepts vs aberrant beliefs. Consistent with previous findings, hallucination-proneness conferred an advantage using prior knowledge to perceive image gist but, contrary to predictions, did not confer disadvantage perceiving image details. Predominant hallucination-proneness actually conferred advantages perceiving both image gist and details, consistent with reliance on highly detailed perceptual knowledge. Delusion-proneness, and especially predominance of delusion-proneness over hallucination-proneness, conferred disadvantage perceiving image details but not image gist, though evidence of specific impairment of detail perception was preliminary. We suggest this is consistent with reliance on abstract, belief-like knowledge. We posit that phenomenological variability in psychotic experiences may be driven by variability in the type of knowledge observers rely upon to resolve perceptual ambiguity.

## Introduction

Hallucinations and delusions can be modeled within a predictive processing framework, in which perceptions and beliefs represent the brain’s best inference about the causes of its sensory inputs.^1–3^ This framework posits that sensation is inherently ambiguous; the brain must compare sensory measurements to predictions, akin to “perceptual hypotheses,”^4,5^ drawn from preexisting knowledge and infer the most likely cause of those sensations. The relative influences of sensory evidence and prior knowledge in this integration are determined by their reliabilities^6^: when sensory information is unreliable, predictions should be weighted more strongly, and vice versa. The reliabilities of sensory information and prior knowledge also shape learning. Disagreement between predictions and sensory inputs generates “prediction errors” that could reflect meaningful changes in environmental states necessitating new learning, ie, changing one’s predictions by updating internal models.^7,8^ Importantly, however, learning should be scaled to the reliability of information sources, with large changes in internal models taking place only when prediction errors are reliable.^9–11^

Psychotic experiences like hallucinations and delusions may arise when reliability weighting of information sources goes awry, causing perceptions and beliefs to diverge from objective reality.^12–14^ Within this framework, hallucinations can be modeled as false inferences, caused by overweighting the reliability of predictions.^15–18^ Delusions may be considered internal models that misrepresent statistical regularities in the environment and could arise through inappropriate learning from unreliable prediction errors.^19^

Such models of psychotic phenomena in patients and psychosis-prone people have been tested by manipulating both prior knowledge and sensory input.^20–22^ In a previous study, in which we kept sensory input constant while manipulating prior knowledge, individuals at high risk of clinical psychosis showed a shift toward greater influence of prior knowledge.^21^ This shift was measured as an advantage in discriminating ambiguous images that contained an embedded figure, the perception of which was facilitated by experimentally provided prior knowledge.^21^ The advantage was also present in healthy individuals scoring highly on scales of aberrant perceptions and aberrant beliefs, though more associated with the former than the latter.

**Fig. 1.**
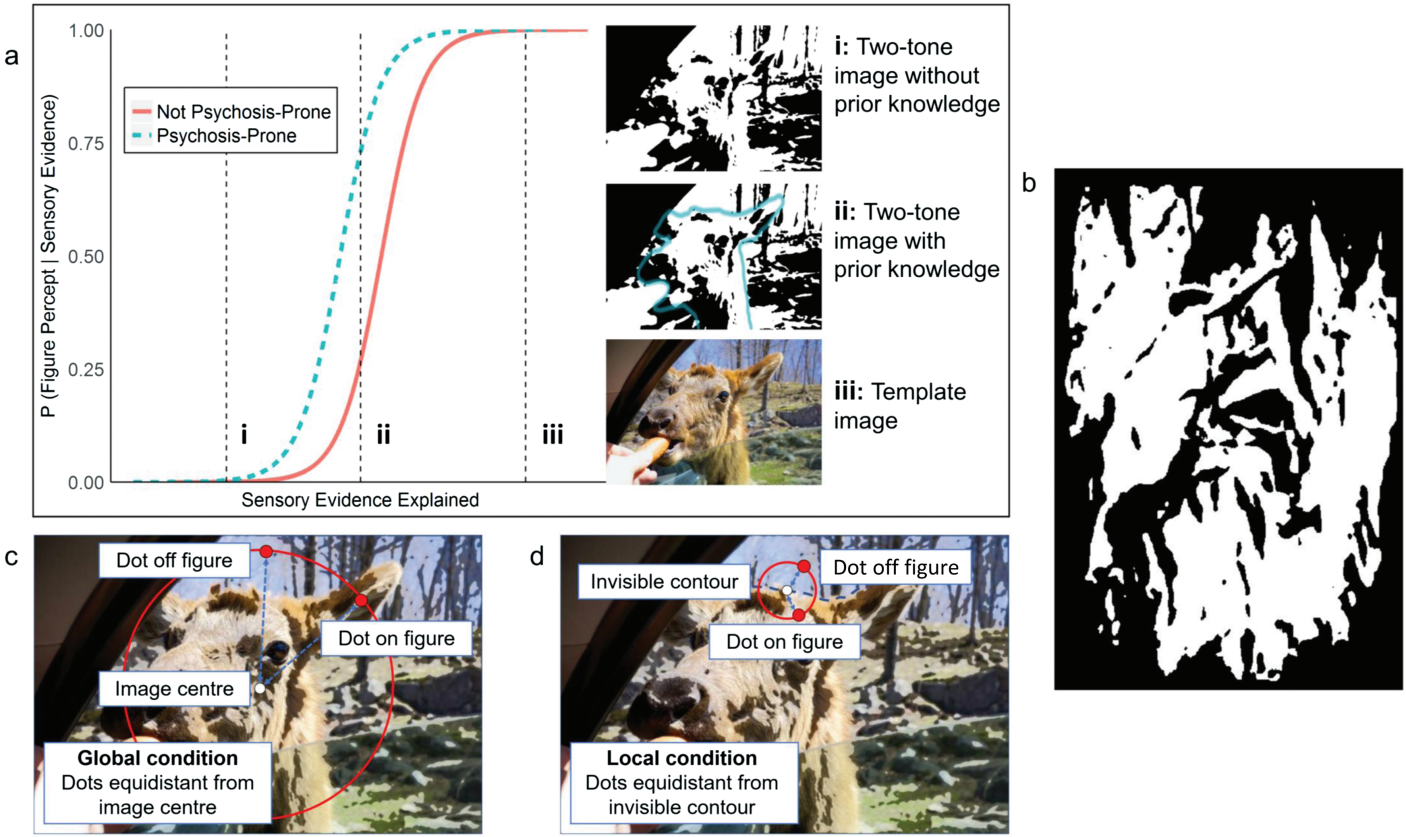
(a) Theoretical function determining probability of generating percepts based on sensory evidence explained by predictions (perceptual hypotheses). At (i), no predictions explain enough evidence in a two-tone to generate a percept. At (iii), templates are recognized using existing prior knowledge. At (ii), prior knowledge increases probability of generating percepts.^23^ Generating a percept when less evidence is explained (leftward-shifted function), which we hypothesized occurs in early psychosis and psychosis-proneness, might readily generate percepts that sometimes misrepresent the environment, giving rise to anomalous inferences. (b) An example of a two-tone image used in the study (see figure 2c for template). (c) Global condition. (d) Local condition.

While this specificity is in line with previous suggestions that hallucinations are a consequence of an increased influence of prior knowledge on perception,^15–18^ it provides no further detail about the underlying computational mechanisms. In the current study, we tested the hypothesis that this shift could be explained by more flexible fitting of predictions to sensory data, such that percepts are generated based on a weaker match between predictions and sensory inputs (figure 1a). This process could maintain stable percepts when sensory evidence is unreliable but might come with the cost of tolerating greater mismatch between predictions and inputs, predisposing to false/inaccurate percepts. In extremis, we consider this a model of hallucinations and aberrant perceptions: a marked dissociation of percepts (and their implications) from sensory evidence.

## Methods

### Manipulating Prior Knowledge in Perception

**Fig. 2.**
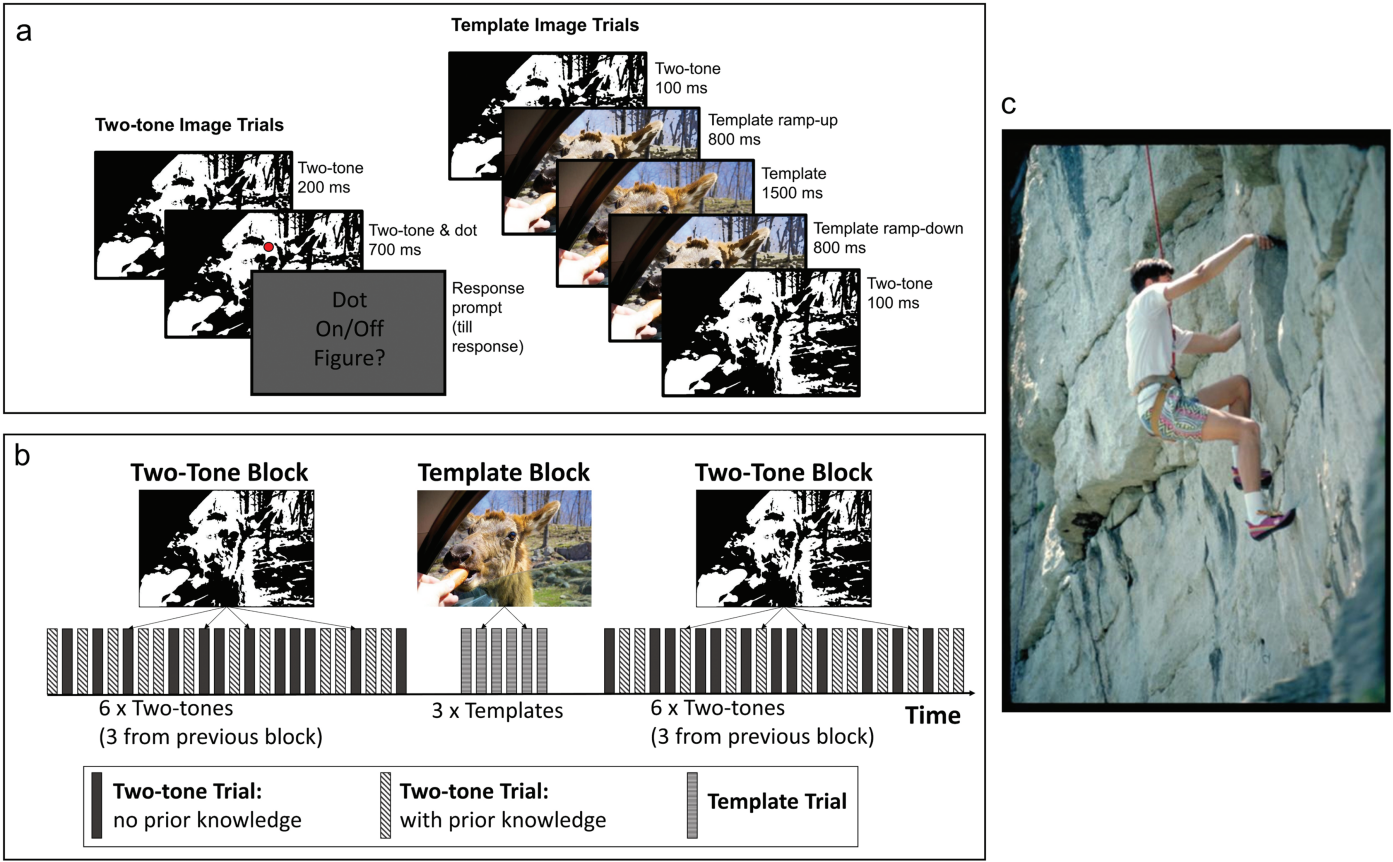
(a) Observers discriminated whether a dot was on/off a figure embedded in two-tone images in “two-tone trials.” Observers gained prior knowledge about two-tones from natural images in “template trials.” (b) Trials were grouped into two-tone and template blocks. Two-tone blocks contained 24 trials with 6 two-tones, each presented 4 times (all combinations of dot on/off, Global/Local condition). Per two-tone block, observers had viewed templates for 3 images (Post-Template) but not the others (Pre-Template). Pre-template and Post-Template two-tones were interleaved. Template blocks contained presented 3 templates, corresponding to Pre-Template two-tones from the preceding block. There were 30 two-tone/template images and 10 of each block. (c) An example of a template image that corresponds to the two-tone in figure 1b.

We investigated the influence of prior knowledge on perception of figures (humans/animals) embedded in “two-tone” images, generated by binarizing natural images around luminance thresholds (figure 1b). Two-tone images were near-impossible to disambiguate in isolation. However, observers could readily generate rich percepts of embedded figures after gaining prior knowledge of image content, provided by viewing the natural image from which a two-tone was generated (the “template” image, figure 2c). For stimuli details, see online supplementary material.

### Task Design

The task had 2 conditions. The “Global” condition (figure 1c) probed the generation of percepts regardless of their accuracy and required knowledge of global image gist. The “Local” condition (figure 1d) probed the generation of percepts with accurate local details. On each trial, observers discriminated whether a small dot on a two-tone was on or off any embedded figures. For each two-tone and each condition, paired possible dot locations were manually chosen, one on-figure and one off-figure.

Global condition locations were equidistant from the image center, far from figure edges and, ideally, separated by patches demarcating figure edges. On-figure/off-figure discriminations should thus be possible even from inaccurate percepts.

Local condition locations were equidistant from a figure contour that was present in the template (identified using an edge detection algorithm) but invisible in the corresponding two-tone. Dot locations were very close on either side of this contour, so on-figure/off-figure discriminations required percepts with accurate fine details.

Observers made the same discriminations before and after gaining prior knowledge from templates on “template trials” (“**Pre-Template**” and “**Post-Template**,” respectively, figure 2a). The same images were used in the Global and Local conditions; furthermore, conditions were interleaved and counterbalanced for within-sequence order effects. Identical **Pre-Template** and **Post-Template** trials allowed us to isolate the effects of prior knowledge on perception by examining within-subject change in performance after seeing the template image (figure 2b). For full details of task design, see online supplementary material.

We predicted that psychosis-prone observers would generate percepts readily but inaccurately, manifesting as advantage in the Global condition and disadvantage in the Local condition.

### Observers

Forty observers who self-reported no current or past mental illness were recruited via university and departmental e-mail lists at the University of Cambridge. The study was approved by the Cambridge Psychology Research Ethics Committee (Reference PRE.2013.31). We measured 2 dimensions of psychosis-proneness with self-report questionnaires. Delusion-proneness was measured with the Peters Delusions Inventory (Brief) (PDI).^24^ Hallucination-proneness was measured with the Cardiff Anomalous Perceptions Scale (CAPS).^25^ The total score (sum of subscales) for each instrument was used.

### Outcome Measures

Objective ability to perform on-figure/off-figure discriminations in each condition was calculated using discriminability index (*d′*), derived from signal detection theory (see online supplementary material for detail). Critically, changes in *d′* could arise from a greater ability to use prior knowledge to extract information from two-tones or from more strategic behaviors like waiting longer to collect information before responding, which might not be related to the use of prior knowledge. We distinguished these possibilities using the drift-diffusion model (DDM), a widely used computational model of perceptual decision-making.

Briefly, the DDM treats two-alternative decision-making as the noisy accumulation of evidence toward 2 decision boundaries over time. The rate of evidence accumulation, parameterized as “drift rate” (*v*), indicates the efficiency of information extraction. Increasing drift rate improves accuracy while speeding decision-making. The information needed to make decisions is indicated by the distance between decision boundaries, parameterized as “decision threshold” (*a*). Increasing decision threshold improves accuracy while *slowing* decision-making. We predicted psychosis-proneness would be associated with more efficient prior knowledge-dependent information extraction from two-tone images, reflected in drift rate, but not with differences in decision threshold. A hierarchical Bayesian DDM was fit to reaction time data using the “HDDM” package^26^ (see online supplementary material for details).

### Statistical Analyses

We investigated effects of conditions and template exposure on *d′*, drift rate, and decision threshold using 2 × 2 factorial ANOVAs with factors “Template-Exposure” (Pre-Template/Post-Template) and “Condition” (Global/Local). Follow-up comparisons were performed with paired Welch’s *t* tests with the Holm correction for control of family-wise error.

Associations with hallucination-proneness and delusion-proneness were investigated using linear regressions predicting the change in *d′* (Δ*d′*), drift rate (Δ*v*), and decision threshold (Δ*a*) caused by gaining prior knowledge through template exposure. Planned univariate analyses were conducted, testing the predicted Global-advantage and Local-disadvantage associated with absolute levels of psychosis-proneness. Planned multiple regression analyses were conducted to test whether the effect was more specifically associated with anomalous perceptions vs anomalous beliefs, predicted on the basis of previous results^21^ (see online supplementary material for details).

We report uncorrected *P* values and investigate the robustness of results using Benjamini-Hochberg step-up correction for 5% false discovery rate over all 36 comparisons with CAPS/PDI.

## Results

Two observers were excluded from the analysis: one saw the stimuli in a previous study; another misunderstood the instructions.

The CAPS and PDI showed positive skew that was corrected to normality by square-root transformation. Hallucination-proneness and delusion-proneness correlated (Pearson *r* = .67, *t*_(36)_ = 5.42, *P* < .001).

### Perceptual Performance

**Fig. 3.**
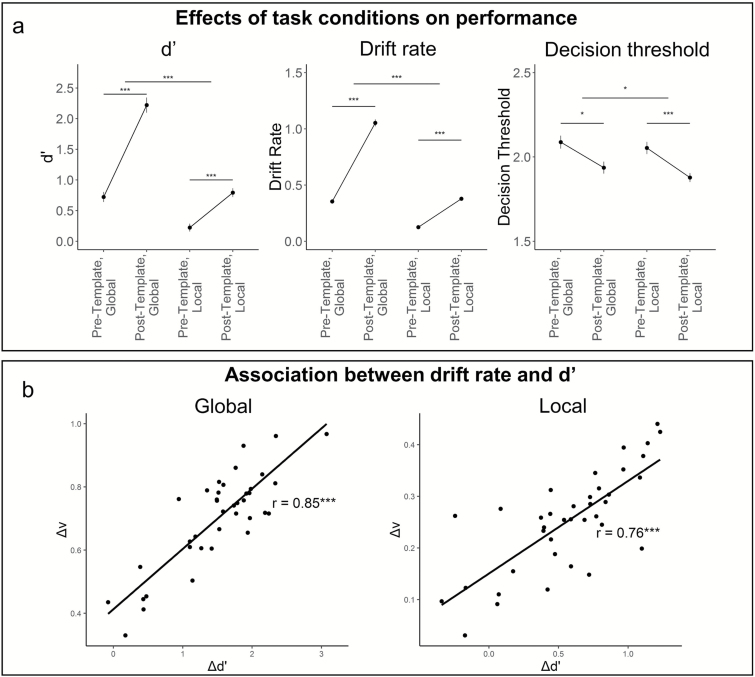
(a) Mean (SE) discriminability (*d′*), drift rate (*v*), and decision threshold (*a*) across task conditions. Template prior knowledge allowed participants to extract evidence faster (higher drift rate, *v*) and make perceptual decisions based on less evidence (lower decision threshold, *a*), with an overall improvement in discrimination (higher *d′*). (b) Δ*d′* and Δ*v* correlated in both conditions, supporting that prior knowledge improved performance by enhancing extraction of information from two-tone images.

Prior knowledge facilitated perception of figures embedded in two-tone images (figure 3a; Template-Exposure main effect: *F*_(1, 40.66)_ = 138.64, *P* < .001), shown in both the Global (*t*_(37)_ = 6.43, *P*_Holm_ < .001, *D* = 2.11) and Local (*t*_(37)_ = 8.44, *P*_Holm_ < .001, *D* = 2.77) conditions. The Global condition was easier (Condition main effect: *F*_(1, 35.31)_ = 120.39, *P* < .001), with higher *d′* Pre-Template (*t*_(37)_ = 6.43, *P*_Holm_ < .001, *D* = 2.11) and Post-Template (*t*_(37)_ = 15.68, *P*_Holm_ < .001, *D* = 5.16), which is consistent with the Global condition requiring only coarse percepts and the Local condition requiring detailed percepts. The magnitude of improvement with template exposure was greater in the Global than the Local condition (Template-Exposure * Condition interaction: *F*_(1, 8.18)_ = 27.89, *P* < .001), confirmed by follow-up comparison (*t*_(37)_ = 8.66, *P*_Holm_ < .001, *D* = 2.85). We interpreted these expected findings as evidence that the task conditions were working as intended.

### Prior Knowledge Facilitated Perceptual Discrimination by Improving Evidence Extraction

Prior knowledge increased the efficiency of information extraction, supported by higher drift rate (Template-Exposure main effect: *F*_(1, 148)_ = 430.5, *P* < .001), evident in Global (*t*_(37)_ = 19.30, *P*_Holm_ < .001, *D* = 6.35) and Local (*t*_(37)_ = 20.98, *P*_Holm_ < .001, *D* = 6.90) conditions (figure 3a).

Information was extracted more efficiently about image gist than about precise details, indicated by higher Global than Local drift rate (Condition main effect: *F*_(1, 148)_ = 477.0_,_*P* < .001), evident in both Pre-Template (*t*_(37)_ = 11.64, *P*_Holm_ < .001, *D* = 3.83) and Post-Template (*t*_(37)_ = 18.29, *P*_Holm_ < .001, *D* = 6.02). Prior knowledge improved information extraction about gist more than about precise details (Template-Exposure * Condition interaction: *F*_(1, 148)_ = 104.8, *P* < .001, follow-up: *t*_(37)_ = 14.11, *P*_Holm_ < .001, *D* = 4.63) (figure 3a).

Prior knowledge caused observers to make decisions based on less evidence, indicated by lower decision threshold (Template-Exposure main effect: *F*_(1, 148)_ = 22.490, *P* < .001), evident in both Global (*t*_(37)_ = 5.124, *P*_Holm_ < .001, *D* = 1.68) and Local (*t*_(37)_ = 7.85, *P*_Holm_ < .001, *D* = 2.58) conditions (figure 3a).

Prior knowledge was therefore only likely to improve on-figure/off-figure discriminations by increasing efficiency of information extraction, rather than observers waiting to accumulate more evidence. Accordingly, Δ*d′* and Δ*v* correlated in the Global (*r* = .85, *t* = 9.56, *P* < .001) and Local (*r* = .76, *t*_(36)_ = 57.04, *P* < 0.001) conditions. There was no association between Δ*d′* and Δ*a*.

### Effects of Hallucination-Proneness and Delusion-Proneness on Perceptual Performance

#### Univariate Analyses.

**Fig. 4.**
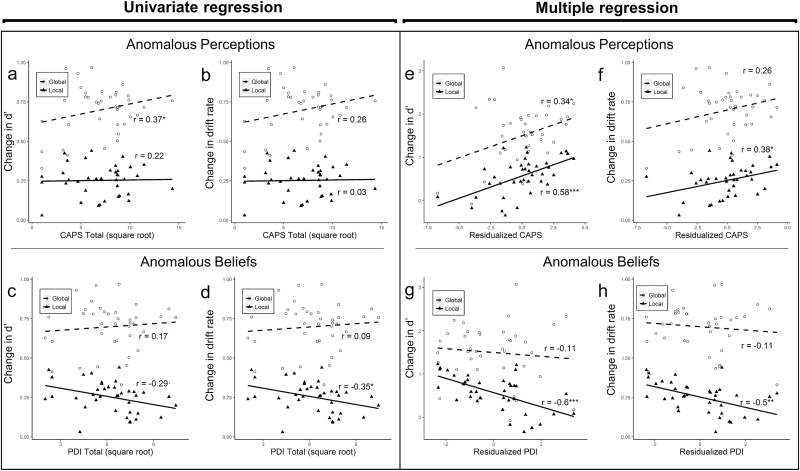
Univariate regressions showed hallucination-proneness (CAPS) was associated with greater improvement in discriminability (Δ*d′*) with template exposure (a) but this was not reflected in change in drift rate (Δ*v*) (b). Delusion-proneness trend-predicted lower Δ*d′* (c) and predicted lower Δ*v* in the Local condition (d). Multiple regressions showed a different pattern of results. Predominant hallucination-proneness predicted greater Δ*d′* in the Global condition (e) but also greater Δ*d′* and Δ*v* in the Local condition (e and h). In contrast, predominant delusion-proneness predicted lower Δ*d′* and Δ*v* in the Local condition (g and h). *R*-values are equivalent correlations. CAPS, Cardiff Anomalous Perceptions Scale.

Consistent with our previous work, hallucination-proneness (CAPS) predicted advantage using prior experience to perceive the gist of figures in two-tones, indicated by association between hallucination-proneness and Global Δ*d′* (figure 4a, *t* = 2.382, *P* = .023, *D* = 0.79, *r*_equiv_ = .37). This was not reflected in Δ*v* (figure 4b). Contrary to predictions, hallucination-proneness was not associated with disadvantage discriminating specific contours, showing no relationship with Δ*d′* or Δ*v* in the Local condition (figures 4a and 4b).

There was a trend toward the hallucination-proneness advantage being specific to gist perception, indicated in difference in Δ*v* across Global and Local conditions (*t* = 1.853, *P* = .072, *D* = 0.62, *r*_equiv_ = .3). This effect was not apparent in Δ*d′.* Together, these results suggested that hallucination-prone healthy observers’ enhanced ability to readily generate template-derived percepts of two-tone images (as evidenced by their advantage in the Global condition) did not come at the cost of an inability to perceive specific details.

We found evidence for the predicted psychosis-associated disadvantage in perceiving image details associated only with delusion-proneness, which, in the Local condition, predicted smaller Δ*v* (figure 4d, *t* = −2.278, *P* = .029, *D* = 0.76, *r*_equiv_ = −.35) and trend-predicted smaller Δ*d′* (figure 4c, *t* = −1.83, *P* = .076, *D* = −0.61, *r*_equiv_ = −.29). Higher delusion-proneness also predicted larger differences in discrimination of image gist and details, indicated by difference in Δ*d′* across conditions (*t* = 2.32, *P* = .026, *r*_equiv_ = .36, *D* = 0.77). Delusion-proneness showed no relationship with Global condition Δ*d′* or Δ*v* (figures 4c and 4d).

#### Multiple Regression Analyses.

We observed striking and opposing effects of the composition of psychotic phenomena in terms of delusion-proneness and hallucination-proneness on perceptual inference.

Observers who were more hallucination-prone than delusion-prone readily generated percepts of figures embedded in two-tone images with a high degree of accurate image detail. Predominant hallucination-proneness (CAPS, controlling for PDI) conferred moderate advantage using prior knowledge to perceive image gist, shown by greater Δ*d′* in the Global condition (figure 4e, *t* = 2.17, *P* = .037, *D* = 0.72), though not reflected in Δ*v* (figure 4f). Importantly, predominant hallucination-proneness conferred large advantage using prior knowledge to perceive image details, shown by greater Δ*d′* and Δ*v* in the Local condition (Δ*d′*: figure 4e, *t* = 4.23, *P* < .001, *D* = 1.41; Δ*v*: figure 4f, *t* = 2.43, *P* = .02, *D* = 0.81), suggesting generation of detailed percepts that were highly faithful to the template information. This advantage was not specific to one condition, with no difference in Δ*d′* or Δ*v* across Global and Local conditions.

In contrast, we found preliminary evidence that observers who were more strongly delusion-prone than hallucination-prone could discriminate a figure’s general location but poorly discriminated precise figure contours. Predominant delusion-proneness (PDI, controlling for CAPS) predicted neither advantage nor disadvantage perceiving gist, with no association with Global condition Δ*d′* or Δ*v* (figures 4g and 4h). However, predominant delusion-proneness predicted large disadvantage in the Local condition (Δ*d′*: figure 4g, *t* = −4.48, *P* < .001, *D* = −1.51; Δ*v*: figure 4h, *t* = −3.43, *P* = .002, *D* = −1.14). There was a trend toward specific Local condition impairment in Δ*d′* across the conditions (*t* = 1.72, *P* = 0.093, *D* = 0.57), though not evident in Δ*v*, suggestive of condition specificity.

Importantly, there were no associations between change in Δ*a* with template exposure and either hallucination-proneness or delusion-proneness in univariate or multiple regressions, supporting our prediction that psychosis-proneness would affect perceptual discrimination by modifying ability to use prior knowledge to extract information from two-tone images.

#### Multiple Testing and Post Hoc Tests.

The multiple regression associations were the most robust; Δ*d′* advantage associated with predominance of hallucination-proneness and disadvantage in Δ*d′* and Δ*v* associated with predominance of delusion-proneness remained significant when corrected for 5% false discovery rate. Our other results did not survive this correction and should thus be considered preliminary.

Given that we only found trend-level evidence of condition-specific effects associated with predominance of anomalous beliefs, we include post hoc analyses in the online supplementary material showing consistent results with conditions collapsed.

## Discussion

We tested the hypothesis that psychosis-proneness in a nonclinical sample would entail a computational shift in perceptual inference toward generating percepts based on poorer fit between sensory inputs and predictions derived from prior knowledge. Psychosis-proneness should thus predict better extraction of coarse perceptual gist in situations that heavily rely on predictions, but an impairment in extracting fine perceptual details. Though partly borne out, our results demand a more complex explanation. First, in keeping with our prediction and with previous results,^21^ hallucination-proneness was associated with a greater ability to use prior knowledge to discriminate figures embedded in ambiguous images, consistent with the idea that anomalous perceptions arise from a changed integration of sensory evidence with top-down predictions.^13,17,27^ However, hallucination-proneness did not predict inaccurate perception of image details. Indeed, when we considered within-individual balance between hallucination- and delusion-proneness, we found that higher levels of aberrant perception in the context of lower levels of aberrant beliefs were associated with better perception of both image gist and fine details. Critically, diffusion-drift modeling showed that this improvement was not due to changes in decision threshold, indicating strategic differences, but was driven by more efficient visual extraction of evidence. This suggests that hallucinations arise from over-reliance on top-down predictions. Contrary to expectations, prior knowledge supported visual extraction of highly detailed perceptual information in hallucination-prone observers with lower anomalous beliefs.

Conversely, delusion-prone observers, and particularly those with higher aberrant beliefs but lower aberrant perceptions, were relatively disadvantaged using prior knowledge to discriminate embedded figures. There was preliminary evidence that this was specific to discriminating those figures’ precise details vs their general locations. This disadvantage was driven by less efficient evidence extraction, rather than changing decision thresholds., Neither absolute delusion-proneness or delusion-proneness relative to hallucination-proneness impaired delusion-prone observers in using prior knowledge to visually extract information pertaining to the images’ broad meanings.

These findings, though complex, are explicable within a processing hierarchy ascending from concrete, unimodal sensory inputs to more abstract, belief-like levels.^28,29^ Within this framework, a computational shift toward over-reliance on prior knowledge in perceptual inference can explain both the advantage held by predominantly hallucination-prone and the disadvantage befalling delusion-prone observers (especially those with low hallucination-proneness). Critically, these effects might arise from overly influential predictions originating from different levels of the processing hierarchy.

Predominantly hallucination-prone observers might preferentially encode and use prior knowledge gained from the template images as perceptual information, conferring an overall advantage discriminating two-tones’ embedded figures. While advantageous in this experiment, under natural viewing conditions this might induce tendencies to interpret ambiguous sensory information using perceptual hypotheses, predisposing to aberrant generation of percepts and, in extremis, hallucinations.

Conversely, predominantly delusion-prone observers might largely encode template prior knowledge as more abstract, belief-like information at upper hierarchical levels. Template knowledge encoded or used at these levels might have sufficient information to support inference on the coarse gist of two-tone images but be of limited use when inferring specific details, as low-level features of the template might be summarized at the higher level without being specifically encoded. The details of resultant percepts may therefore be subtly inaccurate. This would explain preserved performance in the Global condition and poor performance in the Local condition. We suggest two ecological manifestations of this computational shift. Firstly, observers might interpret ambiguous sensory information by invoking higher-level beliefs, manifesting bizarre or delusional appraisals of events. Secondly, observers may be insensitive to fine perceptual details, particularly when not consistent with high-level beliefs. Percepts would effectively be sculpted to conform to expectations, possibly manifesting as false inferences, eg, wrongly inferring intentions of others from subtle facial expressions.

Central to our account is the proposed greater influence of prior knowledge that might arise to compensate for unreliability in signaling of low-level sensory information in the visual system. Such unreliability is supported by the large body of evidence showing dysfunction of early visual processing in psychotic disorders^30^ and some evidence for similar visual impairments associated with schizotypal personality.^31^ These deficits may result in the outputs of early processing being noisier and less well structured, causing ambiguity in perceptual inference and prompting adaptive reliance on top-down predictions.

We consider these conclusions preliminary and requiring replication because we used a relatively small sample size, found unexpected results and some findings failed false discovery rate correction. We found only partial evidence for condition-specific impairment associated with delusion-proneness.

Our account critically suggests that it is the *type* of overly influential prior knowledge, such as perceptual vs abstract knowledge, that shapes the content of psychotic phenomena. This is agnostic to the exact algorithmic use of that prior knowledge.^32^ Bayesian accounts of predictive coding would posit that overly influential predictions, akin to downweighting predictions errors, cause perceptual inference to conform to expectations.^13,16,27^ Within alternative accounts like the circular inference model, excitatory top-down predictions might be mistaken for sensory evidence and reverberate, inducing false inferences.^14^,^33^

These findings provide a starting point for understanding the considerable heterogeneity of positive psychotic phenomena, observed in clinical samples and the general population but poorly addressed by some current theoretical models (though see^14^). Isolated anomalous perceptions or beliefs may represent compensatory changes in using prior knowledge at a single locus of a processing hierarchy, eg, aberrant use of perceptual or abstract knowledge. Why then would delusions and hallucinations co-occur? Concurrence of both phenomena may occur when adaptation at a single level is insufficient to resolve unreliability in sensory inputs and the early outputs of sensory processing, leading to a propagation of atypicalities in the use of prior knowledge throughout the processing hierarchy. Indeed, epidemiological evidence suggests that psychotic phenomena become clinically relevant as anomalous perceptions are complicated by anomalous beliefs.^34^ Using a nonclinical sample limits our ability to generalize these findings to psychotic disorders. However, whether our account of the mechanisms of nonclinical psychotic phenomena extends to clinical psychotic symptoms is readily testable.

To conclude, our results shed light on the emergence and persistence of two seemingly distinct experiences, anomalous perceptions and anomalous beliefs. We put forward the notion that these phenomena could arise from a common computational mechanism, the over-reliance on prior knowledge in the generation of percepts, expressed at different levels of the information processing hierarchy.

## Supplementary Material

Supplementary data are available at *Schizophrenia Bulletin* online.

Supplement_Material

## Funding

This study was supported by Wellcome Trust (grant number WT095692MA) and The Bernard Wolfe Health Neuroscience Fund (to P.C.F.).
