# Supplementary material for: Anomalous Perceptions and Beliefs Are Associated With Shifts Toward Different Types of Prior Knowledge in Perceptual Inference
Source: Schizophr Bull. 2017 Dec 27;44(6):1245–53. doi: 10.1093/schbul/sbx177 (PMC6192467; doi:10.1093/schbul/sbx177)
Supplement: Supplement_Material [file sbx177_suppl_supplement_material.doc]

# Supplementary material

## Stimuli

The stimulus set of two-tone images was expanded and optimised from our previous study^1^ by combining existing two-tone images with novel images, made by binarizing natural images of people and animals around luminance thresholds. All stimuli were then piloted and validated by 4 observers who did not take part in the main experiment. These observers viewed a set of two-tone images, reported what they could see and rated the image for clarity from 1-5, before and after viewing the corresponding template images. 73 suitable images with low clarity ratings before and high clarity ratings after viewing the template images were used as stimuli for the main experiment. The ideal two-tone image was impossible to disambiguate with no prior knowledge and very clear with prior knowledge.

Different observers view two-tone images differently, with some able to see the figures embedded in them spontaneously. To ensure that observers could not disambiguate two-tone images before the main experiment, the observers viewed the set of two-tone stimuli and freely reported whatever they could see in them. Images in which observers recognised any figure or its approximate location were excluded. 30 stimuli were chosen randomly from the remaining pool.

## Task design

There were two trial types in this experiment: two-tone trials, in which participants made decisions about dots being on or off figures in two-tone images, and template trials, in which participants acquired prior knowledge about two-tone images by viewing their natural image counterparts (Figure 2a). Two-tone trials began with a fixation cross on a grey background, then a two-tone image was ramped up in contrast over 200ms. When the two-tone reached full contrast, the dot appeared. After 100ms, the dot flashed off and on for one frame to help observers localise it. The two-tone and dot were presented at full contrast for 700ms then ramped down in contrast over a further 200ms to be replaced with a text prompting observer to respond. Observers had no time limit on making their decision but were instructed to respond as quickly and as accurately as possible.

On each template trial, a two-tone image was first shown for 100ms. Its template image was superimposed and ramped up in contrast over 800ms to give the appearance of smoothly transitioning from two-tone to template. This aided disambiguation and strengthened the subjective percepts of embedded figures. The template image was displayed at maximum contrast for 1500ms then ramped down in contrast over 800ms to reveal the two-tone image, which was then ramped down over 100ms.

Trial type order (on-/off-figure) and conditions (Global/Local) was counterbalanced for within-sequence effects and stimuli were randomly sorted into that sequence. The same image never appeared more than twice successively. 30 two-tone and template images were used in each main experiment, giving 120 **Pre-Template** and 120 **Post-Template** two-tone trials, 60 per Global/Local condition.

Stimuli were presented using Matlab (Mathworks) and the Psychophysics Toolbox^2–4^. Two-tone and template stimuli were 7:5 rectangular and presented on a 15.6” laptop screen with a 60 Hz refresh rate at a distance of approximately 60cm, subtending approximately 17.5° by 12.6°.

## Observers

40 observers (age range = 18-27y, mean = 22.3y, SD = 2.14y, 25 female) were recruited via online advertisement and student email lists at the University of Cambridge. Observers had not previously seen any of the experimental stimuli, had normal/corrected-normal vision, were not colour-blind and had no past/current psychiatric or neurological illness. Observers were reimbursed for their time and gave written informed consent.

## Outcome measures

### Perceptual Performance

Our primary outcome was *d’*, an objective measure of ability to discriminate between on-figure and off-figure trials, derived from signal detection theory. *d’* was calculated using the following equation:


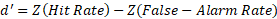
,

where Z is the inverse cumulative standard normal distribution function, ‘Hit Rate’ and ‘False-Alarm Rate’ were the proportions of trials where the dot was correctly and incorrectly identified as on-figure, respectively. Any observers’ hit rates of 1 or false-alarm rates of 0 were adjusted by replacement with 1 – 1/60 1/60 (60 being the number of trials per condition)^5^.

In the Global condition, we adjusted for 3 participants with hit rates equal to 1 and 1 participant with no false-alarms. Removing these observers from analyses did not change any substantive conclusions.

### Diffusion-drift modelling

The diffusion-drift model (DDM) is a widely used mathematical model of information processing that represents evidence accumulation on a single trial as a stochastic random-walk process, where a response is emitted when a certain evidence threshold is reached. Access to higher-quality information is modelled as more rapid accumulation of evidence towards a correct decision and parameterised as ‘drift rate’ (*v*). Increasing drift rate speeds reaction times and increases accuracy. By contrast, requiring more evidence before making a decision can be parameterised as a change in the ‘decision threshold*’*, the distance between evidence thresholds. Increasing decision threshold increases accuracy but slows reaction times.

A hierarchical DDM was fit to RT data using the ‘HDDM’ package^6^. In the hierarchical DDM, model parameters for each subject are treated as random effects drawn from group-level distributions. Parameters at the group and subject level are estimated simultaneously using Bayesian Markov Chain Monte Carlo sampling.

In our model, each DDM boundary reflected a decision that the dot was on or off a figure. The model parameters were drift rate, decision threshold, non-decision time and a bias parameter, indicating tendency to favour on/off decisions. The DDM converged appropriately, assessed by Geweke statistics being less than 2, visual inspection of chain posteriors and simulation of reaction time distributions for each subject.

## Statistical analyses

First, we tested whether the task conditions and template exposure had the intended effects on *d’*, *v* and *a* by entering values on each outcome into 2x2 factorial ANOVAs with factors ‘Template-Exposure’ (Pre-template / **Post-Template**) and ‘Condition’ (Global / Local). Follow-up comparisons were performed with paired Welch’s t-tests with the Holm correction for control of family-wise error.

We next tested whether the effects of seeing the template on *d’* were attributable to changes in *v* or *a* by calculating within-subject change (**Post-Template** – Pre-template) in the Global and Local conditions in *d’*, drift rate and decision threshold (Δ*d’*, Δ*v,* Δ*a* respectively) and calculating Pearson correlations of Δ*d’* with Δ*v* and Δ*a*.

Finally, we tested relationships between dimensions of psychosis-proneness and within-subject change in each outcome variable in the Global and Local conditions separately, as well as the difference in change between the Global and Local conditions.

We investigated effects of psychosis-proneness by separate univariate regressions with hallucination-proneness (CAPS) or delusion-proneness (PDI) predicting the outcome. In our previous study, the advantage in two-tone image perception was more associated with anomalous perceptions than anomalous beliefs^1^. We thus investigated effects of the composition of psychotic phenomena by entering both CAPS and PDI into multiple-regressions predicting each outcome, with the intention of testing whether the effect was more associated with one type of psychotic phenomena over another. We interpreted CAPs, controlling for PDI, as a predominance or excess of anomalous percepts for a given level of anomalous beliefs (and vice versa).

In summary, we ran the following regressions for within-subject change in each outcome (*d’*, *v*, *a*) in each condition (Global, Local, Global-Local difference):


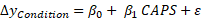


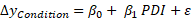


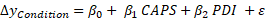


No interactions between CAPS and PDI were considered due to collinearity. In all multivariate regressions, the variance inflation factor was below 4, supporting no collinearity.

## Supplementary Results

### Reaction times

Reaction times for errors and correct answers were analysed separately. Means and standard deviations of reaction times in combinations of correct/incorrect, Global/Local and Pre-Template/Post-Template are shown in Table S1.

Table S1: Mean and standard deviations of reaction times

| Correct/Error | Template Exposure | Condition | Mean Reaction Time (s) | Std. Dev. Reaction Time (s) |
| --- | --- | --- | --- | --- |
| Correct | Pre-Template | Global | 2.44 | 0.89 |
| Correct | Pre-Template | Local | 2.38 | 0.87 |
| Correct | Post-Template | Global | 2.23 | 0.9 |
| Correct | Post-Template | Local | 2.16 | 0.81 |
| Error | Pre-Template | Global | 2.31 | 0.79 |
| Error | Pre-Template | Local | 2.34 | 0.8 |
| Error | Post-Template | Global | 1.93 | 0.67 |
| Error | Post-Template | Local | 2.13 | 0.77 |

Log-transformed trial-by-trial reaction times for correct and incorrect responses were entered into two separate linear mixed-effects models with fixed effects ‘Condition’ (Global/Local) and ‘Exposure’ (Pre-Template/Post-Template) and their interaction and a random effect of subject. Models were estimated using REML with the *lmer* command in R package ‘lme4’. We report regression βs and their 95% confidence intervals as significance tests. Pairwise comparisons for interactions were performed using the *lsmeans* function in packages ‘lsmeans’ and ‘lmerTest’, with the Holm correction for multiple testing.

Reaction times became faster with Template Exposure for both correct (β = -0.11, 95% CI = -0.09 - -0.13) and incorrect (β = -0.08, 95% CI = -0.05 - -0.11) responses. Correct responses were faster in the Global condition than the Local condition (β = 0.09, 95% CI = 0.11 – 0.7), while incorrect responses were faster in the Local condition (β = -0.06, 95% CI = -0.05 - -0.11). There was a significant interaction between condition type and Template exposure for correct (β = 0.08, 95% CI = 0.05 – 0.11) but not incorrect responses (β = -0.03, 95% CI = -0.08 – 0.02).

For correct responses, pairwise comparisons showed that: correct responses were faster after Template exposure in both the Global and Local conditions; post-Template correct responses were faster in the Global condition than the Local condition (all p_Holm_ < 0.001) but there was no difference in pre-Template correct response RTs across conditions.

For error responses, pairwise comparisons showed that: errors were faster after Template exposure in both the Global (p_Holm_ = 0.03) and Local conditions (p_Holm_ < 0.001); pre-Template errors were faster in the Global condition than the Local condition (p_Holm_ < 0.001) but post-Template errors were faster in the Local condition than the Global condition (p_Holm_ = 0.03).

### Post-hoc tests collapsed across Global and Local conditions

We calculated d’ collapsed over both Global and Local conditions. In univariate regressions, absolute hallucination-proneness predicted greater improvement in d’ (t = 2.27, df = 36, p = 0.0296, D = 0.76), while absolute delusion-proneness showed no association. In multiple regressions, predominant hallucination-proneness again predicted greater improvement in d’ (t = 3.48, df = 35, p = 0.001, D = 1.18) while predominant delusion-proneness predicted smaller improvement (t = -2.51, df = 35, p = 0.017, D = 0.84). These effects survived Benjami-Hochberg step-up correction for 5% false discovery rate.

## References

1. Teufel C, Subramaniam N, Dobler V, et al. Shift toward prior knowledge confers a perceptual advantage in early psychosis and psychosis-prone healthy individuals. *Proc Natl Acad Sci*. 2015;112(43):13401-13406. doi:10.1073/pnas.1503916112.

2. Brainard DH. The Psychophysics Toolbox. *Spat Vis*. 1997;10(4):433-436.

3. Kleiner M, Brainard D, Pelli D, Ingling A, Murray R. What’s new in Psychtoolbox-3. *Perception*. 2007.

4. Pelli DG. The VideoToolbox software for visual psychophysics: transforming numbers into movies. *Spat Vis*. 1997;10(4):437-442.

5. Macmillan N, Creelman C. *Detection Theory: A User’s Guide*.; 2004.

6. Wiecki T V, Sofer I, Frank MJ. HDDM: Hierarchical Bayesian estimation of the Drift-Diffusion Model in Python. *Front Neuroinform*. 2013;7:14. doi:10.3389/fninf.2013.00014.
